# Achieving Environmental Sustainability in Africa: The Role of Renewable Energy Consumption, Natural Resources, and Government Effectiveness—Evidence from Symmetric and Asymmetric ARDL Models

**DOI:** 10.3390/ijerph19138038

**Published:** 2022-06-30

**Authors:** Li Yang, Sumaiya Bashiru Danwana, Fadilul-lah Yassaanah Issahaku

**Affiliations:** 1 School of Economics and Management, Anhui University of Science and Technology, No. 168 Taifeng Road, Huainan 232001, China; y321212@163.com; 2 School of Mathematics and Big Data, Anhui University of Science and Technology, No. 168 Taifeng Road, Huainan 232001, China; issakafadil@gmail.com

**Keywords:** environmental sustainability, ecological footprint, renewable energy, government effectiveness, natural resources, Africa, ARDL

## Abstract

This study investigates the symmetric and asymmetric linkages within environmental sustainability proxied by ecological footprint (EFP), natural resources (NRR), renewable energy consumption (REC), urbanization (URB), human capital (HC), and government effectiveness (GE) in 27 African countries divided into two subgroups (ecological deficit countries and ecological reserve countries) over the period 1990 to 2018. The study employs the auto-regressive distributed lag (ARDL) model to investigate the symmetric (linear) effect and the nonlinear auto-regressive distributed lag (NARDL) model to study the asymmetric (nonlinear) effects of the variables on EFP. Results of ARDL show that a 1% increase in REC is projected to reduce ecological footprint by 0.17 and 0.2% in ecological deficit and ecological reserve countries. A 1% increase in NRR is estimated to increase ecological footprint by 0.02% in ecological deficit countries but has no impact on the environment in countries with ecological reserves. Similarly, a 1% rise in GE is estimated to increase EFP by 0.04% in Africa but has no impact on the environment in ecological deficit countries. NARDL estimations decomposed REC into positive (negative) shocks, which show that a 1% increase (decrease) in REC is projected to decrease EFP by 0.16% (0.13%) in countries with ecological reserves. Similarly, a positive (negative) shock in NRR is expected to decrease EFP in ecological reserve countries and increase EFP in ecological deficit countries. Results of the Wald tests prove the existence of long-run asymmetry among the variables. The findings indicate that renewable energy consumption enhances environmental quality, while economic growth and natural resource rents reduce environmental quality in Africa over the sampled period.

## 1. Introduction

The concept of sustainability requires that the production of goods and services fulfills present demands without jeopardizing the potential to satisfy the needs of future generations [1]. The environment is a finite resource; a healthy environment benefits the ecosystem and all life. Therefore, to sustain the planet, our ecosystem, and all life on it, it is critical that environmental resources be appropriately managed and preserved. In recent years, fighting environmental degradation has been a key priority for advanced and emerging countries [2,3]. Environmental degradation has posed a danger to the economic well-being of the entire world, as it is linked to the success of various macroeconomic factors [4]. Over the last two decades, research has contributed significantly to society by demonstrating how human beings influence global ecosystem changes [5,6,7,8]. The effects of human activities originate from the use of the environment to produce goods for consumption and as a receptacle for waste [9]. Danish, Ulucak, and Khan [10] consider unsustainable manufacturing and consumption behaviors important pollution and environmental degradation factors. With the increasing impact of global warming and climate change on the environment and human beings, policy efforts are still insufficient to change unsustainable resource use practices. Environmental challenges throughout the world are quickly worsening, and no nation or area is spared from the consequences of climate change [11].

Located between Europe and Asia, Africa is the world’s second-most populous and biggest continent. Despite countless worldwide crises, Africa has grown at a rate of 5% per year on average during the last decade [12]. This quick expansion has sparked numerous conspiracy theories about Africa, resulting in its notable change from a continent of instability, violence, and famine to commercial opportunity and progress [12]. Climate change is becoming a more significant issue in Africa, one of the most susceptible continents to global warming [13]. Less than 10 percent of the world’s greenhouse gas emissions come from Africa [13]. Africa’s limited adaptation ability renders it so much more susceptible to the effects of climate change [14], notwithstanding its modest contribution to global greenhouse gas emissions [15]. The changing climate is already taking a toll on Africa. Africa appears to be among the world’s severely impacted areas, with regular occurrences of global-warming-related developments [16]. Water shortages, flooding, hot temperatures, and cyclones have affected infrastructure profoundly and disrupted the lives of millions of families [13]. According to the United Nations (UN), African nations will be badly impacted by increasing temperatures in the following decades unless efforts are made to mitigate global warming.

In Africa, natural resource extraction is a significant cause of environmental degradation [17]. Africa has a diverse range of natural resources. The continent produces a substantial amount of the world’s natural resources and earns tremendous income from natural resource exports [18]. Unsustainable resource use and extraction, coupled with mismanagement and weak policy enactment [18], have contributed to the increasing rate of deforestation [19] and climate change [6,20]. The constant decline of forests has made it increasingly difficult for Africa to combat climate change [21]. The Food and Agricultural Organization (FAO) of the United Nations reports that Africa lost 4.4 million hectares of forest yearly from 2015 to 2020 [22], double the global average. While deforestation increases agricultural land, it also rips the land of vital nutrients, resulting in only short-term crop production. According to UNEP, over 50% of Africa’s eco-regions have lost 50% of their land to degradation, agriculture, or urbanization. Over 2 million square kilometers of protected areas remain in Africa. Issues linked with oil and mineral exploitation, unregulated fishing, inefficient management of tropical forests, and coastline expansion continue to plague the continent’s coastal cities. Many species’ habitats are being destroyed by exploiting forest trees for shelter and charcoal. The utmost task of preserving the environment and ensuring the replenishment of resources necessitates a worldwide collaboration between governments, local communities, and the general public. In this context, sustainability necessitates those natural resources are not used beyond their regenerative capacity [23].

African governments attempt to cut carbon emissions and establish sustainable ecosystems in their nations, as global climate change becomes more severe. However, environmental sustainability necessitates considering environmental boundaries. These limits specify the highest rate of deterioration that a resource may undergo before being substantially damaged. Environmental laws and policies exist to protect natural resources from being exploited in this way. These laws safeguard the quality of life and economic prosperity while not jeopardizing the well-being of the environment. Environmental regulations may not always minimize environmental deterioration, since their ineffective enforcement can sometimes outweigh their anticipated advantages. While prosperous industrialized nations have shown that strong governance is the source of their better performance, there is significant evidence that inefficient governance impacts Africa’s economic performance. Africa’s poor governance has resulted in poor economic development, often reflected in the weak rule of law and institutions [16]. Studies have shown that good governance and institutions are needed to achieve environmental sustainability targets [24,25,26]. This justifies the inclusion of the function of governance effectiveness in terms of assessing environmental sustainability in Africa. Because political institutions control environmental policy, it is important to incorporate governance effectiveness in the model.

The ecological footprint has lately gained attention from researchers to measure environmental degradation [10,19,27,28,29,30,31]. Initially proposed by Rees [32], the ecological footprint meets all the criteria for an extensive, progressive, and comprehensive assessment of human-caused environmental harm [10,17,33]. The ecological footprint is an all-encompassing measurement of resource use that reveals the ways human consumption exceeds acceptable limits [23,34]. According to the global footprint network, ecological footprint monitors how productive surface areas are used. At the same time, biocapacity tracks the performance of ecological resources [33,35]. Cropland, forest, grazing, fishing, built-up land, and carbon emissions are the six components of this indicator, demonstrating the effects of human activities on the environment. The ecological footprint is acknowledged as the best proxy for environmental deterioration as a policy instrument, since it provides a broader and complete assessment of human-caused strain on the environment [8,31,36]. An ecological deficit will emerge if the ecological footprint is higher than the biocapacity of a region, that is, when natural resources are depleted faster than they are replenished [10,37]. Conversely, there is an ecological reserve when the ecological footprint is lower than biocapacity. The ecological footprint is a valuable indicator of resource sustainability and consumption pattern throughout the world [10].

**Figure 1:**
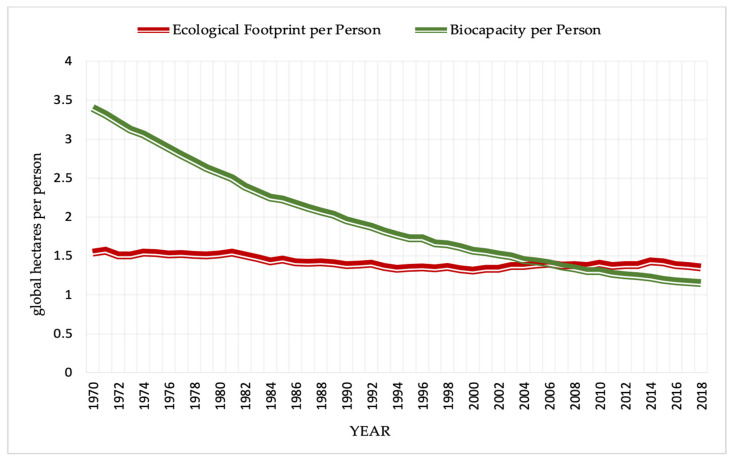
Africa’s ecological footprint and biocapacity per person from 1990 to 2018.

Each African country has its ecological footprint data, even though most countries have similar characteristics. Africa is in an ecological deficit; its production footprints are greater than its biocapacity, implying domestic natural capital is being destroyed by emitting more carbon dioxide than the environment can absorb. Africa’s ecological footprint was 1.35 hectares per person in 2018, which is much less than the world average of 2.8 hectares per person and a biocapacity of 1.15 hectares per person. Gabon, Congo, and the Central African Republic are among the top ten nations globally with the highest biocapacity compared to the population. Africa experienced a constant decline in biocapacity from 1960 until 2014, when an ecological deficit was recorded in 60 percent of countries on the continent [33]. Since then, the ecological deficit has been increasing, and though minimal compared to the world average, it is a cause for concern. The ecological footprint and biocapacity per capita of Africa are illustrated in Figure 1.

Energy generated from fossil fuels has been identified as a significant cause of pollution [1,25,36,38]. Despite being a major cause of pollution, fossil fuels remain the most used source of energy in Africa and the rest of the world [39,40]. In most African countries, the lack of sufficient and reliable energy supplies has been a significant impediment to economic growth [41]. According to a recent assessment by the International Energy Agency, Africa’s energy demand is expected to rise by 50 percent in 2040, and renewable energy has the greatest potential to meet this demand [42]. In this respect, international conventions (the Kyoto Protocol and the Paris Agreement) urge that nonrenewable energy be substituted with renewable energy sources [1,43]. Despite being the continent with the highest solar energy resource globally, Africa generates less than 1 percent of the global total of solar energy [42]. The capacity to leverage the available renewable energy sources has proven to be a big challenge for many African nations [44,45]. Numerous advanced and developing countries have embraced renewable energy sources as viable green energy sources to comply with global environmental conventions. Renewable energy sources derived from natural resources are considered clean energy sources with less negative environmental consequences [46,47,48]. Furthermore, these sources are long-term viable for current and future economic demands [36]. Renewable energy is highly praised for its ability to reduce ecological impacts [47]. Renewable energy consumption has expanded globally due to its environmental advantages [49].

This study contributes to the literature by investigating the symmetric and asymmetric linkages between environmental sustainability, natural resources, renewable energy consumption, urbanization, human capital, and government effectiveness in 27 African countries in two subgroups of countries with ecological deficits and countries with ecological reserves over the period 1990–2018. The study employs the panel nonlinear auto-regressive distributed lag and panel auto-regressive distributed lag (ARDL) models. Unlike previous studies, which used carbon emissions, this study uses ecological footprint as a proxy for environmental sustainability. This choice was made because the ecological footprint is a more comprehensive measure of environmental degradation than carbon emission [1,11,36]. Government effectiveness as a factor influencing ecological footprint is a topic that has not received much attention. Reducing environmental pressures while maintaining economic progress is a dilemma for governments all around the globe [35]. Our research provides new empirical evidence on the role of government effectiveness in environmental protection in African countries with ecological deficits and African countries with ecological reserves. To the best of our knowledge, no empirical study has investigated the symmetric and asymmetric linkages between ecological footprint, natural resources, green energy consumption, urbanization, human capital, and government effectiveness in Africa with subsamples of countries with ecological deficits and countries with ecological reserves.

The findings of this study will help highlight disparities in the approaches to environmental sustainability in each subsample. This study offers projections for the context in these nations over the next several years, providing valuable information to the authorities in charge of addressing future ecological deficits. Limiting the study to one continent helps us notice the differences across countries. The findings will also inform politicians and the public about the need to refocus on initiatives toward achieving environmental sustainability under the Sustainable Development Goals (SDGs), the Kyoto Protocol, the Paris Agreement, and several others. This research informs governments and policymakers on the need to prioritize the quality of policy formulation and implementation and improve governance to increase environmental protection measures.

While there is no denying the work’s importance, it only gives attention to the long- and short-run side of the analysis, while ignoring the nonlinear links across various levels of the dependent variable (ecological footprint) and cross-country linkages. The quantile ARDL model proposed by Cho et al. [50] and the dynamic factor model used in the works of Delgado et al. [51] can be of interest in supplementing and improving the study of environmental sustainability in the African context.

The rest of the paper is structured as follows. Section 2 briefly reviews the literature. Section 3 presents the data and models. Section 4 discusses the empirical results, and Section 5 provides some conclusions, policy recommendations, and limitations of the study.

## 2. Literature Review

### 2.1. Renewable Energy Consumption and the Environment

Renewable energy is produced from naturally existing sources that are automatically replenished [40]. Many governments have made renewable energy one of their primary goals for minimizing environmental deterioration [36,52]. Danish et al. and Qamruzzaman and Jianguo [49,53] attest that renewable energy consumption will provide a means for sustainability and a green economy. In exploring the relationship between green energy consumption (renewable energy) and the environment, Imisi and Philip [54] put forth that renewable energy and nonrenewable energy consumption have an enormous capacity to influence the environment, since they can change the ecological footprint of a country [36]. Salim, Rafiq, and Shafiei [55] found that renewable energy consumption negatively predicted ecological footprint. Khan et al. [25] discovered that using energy from renewable sources rather than fossil fuels is advantageous to the environment, since nonrenewable energy use increases carbon emissions. In a similar study of south Asian countries with data from 1996 to 2019 using the cross-sectional autoregressive distributed lag method (CS-ARDL), Mehmood [26] found that renewable energy reduces carbon emissions substantially. The findings of Mehmood align with Zeb et al. [56] and Khan et al. [4], who also found a negative relationship between carbon emissions and renewable energy consumption. Contrary to many findings, the empirical evidence from Mulali, Solarin, and Ozturk [57] showed an insignificant relationship between renewable energy consumption and environmental degradation. Mulali et al. [58] studied the effect of renewable energy consumption on the ecological footprint in 58 developed and developing countries from 1980 to 2009 using the fixed effect, difference, and system generalized method of moments. Their results indicated that renewable energy production increases ecological footprint in the long run.

There are currently no satisfactory solutions to how renewable energy consumption affects ecological footprint and environmental damage in Africa’s diverse countries. This question concerning the link between renewable energy and the environment requires empirical data to give the necessary knowledge for mitigating climate change, reducing ecological footprints, and implementing more effective energy plans. This research addresses this issue by providing an improved and robust analysis of these interactions in the African context.

### 2.2. Natural Resources and the Environment

Previous literature on natural resource studies primarily concentrates on the relationship between natural resources and economic growth [59,60]. Natural resources are undoubtedly a strong determinant of economic growth [61,62]. The pursuit of high economic prosperity has led to large-scale and unsustainable resource extraction and consumption that have caused damage to the environment [2,4]. For this reason, recent studies are more focused on the environmental impacts of natural resource extraction and consumption. Jahanger et al. [4] studied a panel on 73 developing countries from 1990 to 2016 and concluded that natural resources increase ecological footprints. In a recent study, Sun et al. [62] investigated the asymmetric effect of natural resources on environmental pollution in China’s 30 regions using the GMM system with data from 2000 to 2019. They concluded based on their finding that natural resource has a substantial negative impact on the environment. Over the period 1980 to 2016, Erdoğan et al. [18] found that natural resource use accounts for increases in the ecological footprints of 23 Sub-Saharan African countries. The findings of Ahmad et al. [63] conform to those of Refs [4,18,62]. Ahmed et al. add that advanced technology in restoring natural resources could help reduce environmental degradation and ecological footprints. Negative links between natural resources and ecological footprint resonate with the findings of Sun et al. [64].

A few other studies believe natural resources are good for the environment and reduce ecological footprints. In one such study, Kongbuamai et al. [65] investigated the impact of natural resources in ASEAN countries from 1995 to 2016. They concluded that natural resources improve the quality of the environment in ASEAN countries. In the context of BRICS countries, the findings of Danish et al. [10] conform to those of Kongbuamai et al. when they found that the use of natural resources has a negative relationship with the ecological footprint. Zafar et al. [66] add that natural resource abundance reduces ecological footprint.

### 2.3. Government Effectiveness and the Environment

The function of government in influencing the quality of the environment is highly relevant [26]. The government’s responsibility is to enact and implement policies that encourage the sustainable use of resources and determine better ways of achieving environmentally friendly growth [67]. Only a few studies have looked at the effects of governance on the environment but have produced mixed results. Khan et al. [25] reveal that government effectiveness has not yet achieved the intended level in countries with regard to the environment. As a result, it does not play a sufficient role in maintaining the quality of the environment. According to the findings of Adekunle [16], government effectiveness has a negative relationship with environmental sustainability. Based on the above, it is crucial to provide empirical evidence to show the role of government effectiveness in achieving environmental sustainability.

## 3. Materials and Methods

### 3.1. Data and Variables

The study used panel data from 27 selected African countries from 1990 to 2018, including 17 countries with ecological deficits (Egypt, Mauritius, Algeria, South Africa, Tunisia, Uganda, Morocco, Kenya, Rwanda, Lesotho, Togo, Nigeria, Ghana, Burundi, Malawi, Senegal, and Burkina Faso) and 10 countries with ecological reserves (Gabon, Democratic Republic of Congo, Central African Republic, Namibia, Madagascar, Angola, Cote d’Ivoire, Botswana, Cameroon, and Mali). This study focused on 5 southern African countries, 7 eastern African countries, 4 northern African countries, 8 west African countries, and 4 central African countries. Due to data availability constraints, only 27 African countries were selected for this research. Data for the study were obtained from the World Development Indicators, the Global Footprint Network, and Penn World Table 10.0. A detailed description of the variables is shown in Table 1.

### 3.2. Model Specification

We generate the following equation to study the relationship between environmental sustainability (proxied by ecological footprint) and the other variables.

(1) EFP=f(REC,NRR,GE,GDP,URB,HC)

where *EFP* is ecological footprint, *REC* is renewable energy consumption, *GE* is government effectiveness, *GDP* is gross domestic product, *URB* is urbanization, and *HC* is human capital.

We proceed to express the equation as a function form for empirical analysis, as follows:

(2) $$lnEFP_{it}=\alpha_{0}+\alpha_{1}lnREC_{it}+\alpha_{2}lnNRR_{it}+\alpha_{3}lnGE_{it}+\alpha_{4}lnGDP_{it}+\alpha_{5}lnURB_{it}+\alpha_{6}lnHC_{it}+\mu_{it}$$

where *lnEFP*, *lnREC*, *lnNRR*, *lnGE*, *lnGDP*, *lnURB*, and *lnHC* are the natural logarithms of *REC*, *NRR*, *GE*, *GDP*, *URB*, and *HC*, respectively. Variables are converted to their natural logarithm form to reduce the level of skewness in the data. *i* represent the cross-section of countries used, *t* represents time, and *µ_it_* defines the error term.

#### 3.2.1. The Symmetric Panel PMG ARDL Model

To examine the long- and short-run causalities of ecological footprint, green energy consumption, natural resources, urbanization, human capital, and government effectiveness, the present study employed auto-regressive distributed lag (ARDL) models. The panel ARDL model is more useful when estimating non-stationary panel data because it provides consistent estimates of short-run dynamics and long-rung relationships regardless of the order of integration of the variables. In ARDL estimations, variables can be integrated of order one (I (1)) or a mixture of I (0) and I (1) [12,43,68,69]; none of the variables can be I (2) or more. The versatility of the panel ARDL approach [68] rests in its ability to establish long-run relationships between variables even when conventional cointegration requirements do not apply [70].

The pooled mean group (PMG), mean group (MG), and dynamic fixed effects (DFE) models are the three main approaches for evaluating the ARDL model. On panel data, the PMG, MG, and DFE estimators are known to be effective at detecting the existence of a long-run relationship. Furthermore, these estimators consider variation in the transitions of variable adjustments toward long-run equilibrium. The influence of the independent variables on the dependent variable varies by country due to the unique conditions of each country; the estimated coefficients for each independent variable are likely to be influenced by a substantial heterogeneity bias. The pooled mean group panel model accounts for the unobserved cross-sectional characteristics, while restricting long-run coefficients to be the same, allowing for the variability of short-run coefficients across panels [43,49,70,71]. It is preferable to apply an estimate that allows for the distinction between short-term and long-term dynamics, while accounting for country-specific differences.

For this reason, we use the PMG estimator. The pooled mean group estimation of the ARDL model (*p*, *r*, *s*, *t*, *u*, *v*, *w*), including the long-term relationship between the variables, is as follows

(3) $$\begin{array}{cc} \Delta lnEFP_{it}=\alpha_{0} & +\sum_{d=1}^{p-1}\beta_{id}\Delta lnEFP_{i,t-d}+\sum_{f=0}^{r-1}\gamma_{if}\Delta lnREC_{i,t-f}\\ & +\sum_{q=0}^{s-1}\delta_{iq}\Delta lnNRR_{i,t-q}+\sum_{j=0}^{t-1}\phi_{ij}\Delta lnGE_{i,t-j}\\ & +\sum_{k=0}^{u-1}\varphi_{ik}\Delta lnGDP_{i,t-k}+\sum_{l=0}^{v-1}\psi_{il}\Delta lnURB_{i,t-l}\\ & +\;\sum_{m=0}^{w-1}\lambda_{im}\Delta lnHC_{i,t-m}+\theta_{1}lnEFP_{i,t-1}+\theta_{2}lnREC_{i,t-1}\\ & +\theta_{3}lnNRR_{i,t-1}+\theta_{4}lnGE_{i,t-1}+\theta_{5}lnGDP_{i,t-1}+\theta_{6}lnURB_{i,t-1}\\ & +\theta_{7}lnHC_{i,t-1}+\varepsilon_{i,t}\;\; \end{array}$$

where *i* is the countries (1, 2, 3, 4, 5… 27), and t is the time frame (1990–2018). $\beta_{id}$, $\gamma_{if}$, $\delta_{iq}$, $\phi_{ij}$, $\varphi_{ik}$, $\psi_{il}$, and $\lambda_{im}$ are the short-run coefficients of each country in the sample. *p, r, s, t u, v* represent the optimal time lags. $\varepsilon_{i,t}$ is the error term. The PMG ARDL considers the long-run coefficients of all countries to be the same. The existence of a long-run stochastic trend (cointegration) is established if $\theta_{1}+\theta_{2}+\theta_{3}+\theta_{4}+\theta_{5}+\theta_{6}+\theta_{7}\neq 0$. There is no long-run relationship (cointegration) if $\theta_{1}+\theta_{2}+\theta_{3}+\theta_{4}+\theta_{5}+\theta_{6}+\theta_{7}=0$. Equation (4) presents the long-run model of our study.

(4) $$\begin{array}{cc} \Delta lnEFP_{it}=\mu_{1} & +\sum_{d=1}^{p-1}\theta_{d}\Delta lnEFP_{i,t-d}+\sum_{f=0}^{r-1}\theta_{f}\Delta lnREC_{i,t-f}\\ & +\sum_{q=0}^{s-1}\theta_{q}\Delta lnNRR_{i,t-q}+\sum_{j=0}^{t-1}\theta_{j}\Delta lnGE_{i,t-j}\\ & +\sum_{k=0}^{u-1}\theta_{k}\Delta lnGDP_{i,t-k}+\sum_{l=0}^{v-1}\theta_{l}\Delta lnURB_{i,t-l}\\ & +\;\sum_{m=0}^{w-1}\theta_{m}\Delta lnHC_{i,t-m}+v_{i,t} \end{array}$$

Equation (5) presents the error correction model (ECM), which is used to establish the short-run association between the variables in our study.

(5) $$\begin{array}{cc} \Delta lnEFP_{it}=\alpha_{0} & +\sum_{d=1}^{p-1}\beta_{id}\Delta lnEFP_{i,t-d}+\sum_{f=0}^{r-1}\gamma_{if}\Delta lnREC_{i,t-f}\\ & +\sum_{q=0}^{s-1}\delta_{iq}\Delta lnNRR_{i,t-q}+\sum_{j=0}^{t-1}\phi_{ij}\Delta lnGE_{i,t-j}\\ & +\sum_{k=0}^{u-1}\varphi_{ik}\Delta lnGDP_{i,t-k}+\sum_{l=0}^{v-1}\psi_{il}\Delta lnURB_{i,t-l}\\ & +\;\sum_{m=0}^{w-1}\lambda_{im}\Delta lnHC_{i,t-m}+\omega_{i}ECT_{i,t-1}+\varepsilon_{i,t} \end{array}$$

$ECT_{i,t-1}$ is the error correction term. $\omega_{i}$ is the coefficient of the error term, which is expected to be significant and negative (between the values of 0 and −1). Divergence from the long-run equilibrium affects short-run dynamics. Cointegrated variables have an error correction term generated to recover any information lost during differentiation to achieve short-term dynamics and long-term equilibrium. The ECT predicts the speed at which the dependent variable reverts to equilibrium after a change in any of the other variables.

#### 3.2.2. Panel Nonlinear Autoregressive Distributed Lag (NARDL) Model

The NARDL model is an extension of the ARDL model. Because relationships among the variables are not necessarily linear, nonlinear models are increasingly popular [72]. Compared to the linear model structure, nonlinear techniques give a greater array of perspectives. The nonlinear panel autoregressive distributed lag model (NARDL) developed by Shin et al. [73] was used to quantify possible asymmetries stemming from changes in our independent variables. The nonlinear form of Equation (2) is presented as follows:

(6) $$\begin{array}{cc} lnEFP_{it}=\alpha_{0} & +\alpha_{1}^{+}lnREC_{it}^{+}+\;\alpha_{2}^{-}lnREC_{it}^{-}+\alpha_{3}^{+}lnNRR_{it}^{+}+\alpha_{4}^{-}lnNRR_{it}^{-}\\ & +\;\alpha_{5}^{+}lnGE_{it}^{+}+\alpha_{6}^{-}lnGE_{it}^{-}+\;\alpha_{7}^{+}lnGDP_{it}^{+}+\alpha_{8}^{-}lnGDP_{it}^{-}\\ & +\alpha_{9}^{+}lnURB_{it}^{+}+\alpha_{10}^{-}lnURB_{it}^{-}+\;\alpha_{11}^{+}lnHC_{it}^{+}+\alpha_{12}^{-}lnHC_{it}^{-}+\varepsilon_{it} \end{array}$$

In Equation (6), independent variables are decomposed into negative and positive partial sums to test the nonlinear relationship between positive and negative shocks of the variables. $\alpha_{0}$, $\alpha_{1}^{+}$, $\alpha_{2}^{-}$, $\alpha_{3}^{+}$, $\alpha_{4}^{-}$, $\alpha_{5}^{+}$, $\alpha_{6}^{-}$, $\alpha_{7}^{+}$, $\alpha_{8}^{-}$, $\alpha_{9}^{+}$, $\alpha_{10}^{-}$, $\alpha_{11}^{+}$, and $\alpha_{12}^{-}$ are the parameters, and $lnREC_{it}^{+}$, $lnREC_{it}^{-}$, $lnNRR_{it}^{+}$, $lnNRR_{it}^{-}$, $lnGE_{it}^{+}$, $lnGE_{it}^{-}$, $lnGDP_{it}^{+}$, $lnGDP_{it}^{-}$, $lnURB_{it}^{+}$, $lnURB_{it}^{-}$, $lnHC_{it}^{+}$, and $lnHC_{it}^{-}$ are the long-run vectors that are unknown (yet to be estimated), and $\varepsilon_{it}$ is the error term. The decomposition of variables into their negative and positive shocks is shown below:

(7a) $$lnREC_{it}^{+}=\overset{t}{\underset{k=1}{\sum }}\Delta lnREC_{ik}^{+}=\overset{t}{\underset{k=1}{\sum }}\max\left(\Delta lnREC_{ik,}0\right)$$

(7b) $$lnREC_{it}^{+}=\overset{t}{\underset{k=1}{\sum }}\Delta lnREC_{ik}^{+}=\overset{t}{\underset{k=1}{\sum }}\min\left(\Delta lnREC_{ik,}0\right)$$

(8a) $$lnNRR_{it}^{+}=\overset{t}{\underset{k=1}{\sum }}\Delta lnNRR_{ik}^{+}=\overset{t}{\underset{k=1}{\sum }}\max\left(\Delta lnNRR_{ik,}0\right)$$

(8b) $$lnNRR_{it}^{-}=\overset{t}{\underset{k=1}{\sum }}\Delta lnNRR_{ik}^{-}=\overset{t}{\underset{k=1}{\sum }}\min\left(\Delta lnNRR_{ik,}0\right)$$

(9a) $$lnGE_{it}^{+}=\overset{t}{\underset{k=1}{\sum }}\Delta lnGE_{ik}^{+}=\overset{t}{\underset{k=1}{\sum }}\max\left(\Delta lnGE_{ik,}0\right)$$

(9b) $$lnGE_{it}^{-}=\overset{t}{\underset{k=1}{\sum }}\Delta lnGE_{ik}^{-}=\overset{t}{\underset{k=1}{\sum }}\min\left(\Delta lnGE_{ik,}0\right)$$

(10a) $$lnGDP_{it}^{+}=\overset{t}{\underset{k=1}{\sum }}\Delta lnGDP_{ik}^{+}=\overset{t}{\underset{k=1}{\sum }}\max\left(\Delta lnGDP_{ik,}0\right)\;$$

(10b) $$lnGDP_{it}^{-}=\overset{t}{\underset{k=1}{\sum }}\Delta lnGDP_{ik}^{-}=\overset{t}{\underset{k=1}{\sum }}\min\left(\Delta lnGDP_{ik,}0\right)$$

(11a) $$lnURB_{it}^{+}=\overset{t}{\underset{k=1}{\sum }}\Delta lnURB_{ik}^{+}=\overset{t}{\underset{k=1}{\sum }}\max\left(\Delta lnURB_{ik,}0\right)$$

(11b) $$lnURB_{it}^{-}=\overset{t}{\underset{k=1}{\sum }}\Delta lnURB_{ik}^{-}=\overset{t}{\underset{k=1}{\sum }}\min\left(\Delta lnURB_{ik,}0\right)$$

(12a) $$lnHC_{it}^{+}=\overset{t}{\underset{k=1}{\sum }}\Delta lnHC_{ik}^{+}=\overset{t}{\underset{k=1}{\sum }}\max\left(\Delta lnHC_{ik,}0\right)$$

(12b) $$lnHC_{it}^{-}=\overset{t}{\underset{k=1}{\sum }}\Delta lnHC_{ik}^{-}=\overset{t}{\underset{k=1}{\sum }}\min\left(\Delta lnHC_{ik,}0\right)$$

Taking into consideration the negative and positive partial sum decomposition of our variables, the NARDL model for our study is written as follows.

(13) $$\begin{array}{cc} \Delta lnEFP_{it}=\alpha_{0} & +\sum_{d=1}^{p-1}\beta_{id}\Delta lnEFP_{i,t-d}+\sum_{f=0}^{r-1}(\gamma_{if}^{+}\Delta lnREC_{i,t-f}^{\;\;\;\;\;\;\;\;\;+}+\gamma_{if}^{-}\Delta lnREC_{i,t-f}^{\;\;\;\;\;\;\;\;\;\;\;-})\\ & +\sum_{q=0}^{s-1}(\delta_{iq}^{+}\Delta lnNRR_{i,t-q}^{\;\;\;\;\;\;\;\;\;\;\;+}+\delta_{i}^{-}\Delta lnNRR_{i,t-q}^{\;\;\;\;\;\;\;\;\;\;-})\;\\ & +\sum_{j=0}^{t-1}(\phi_{ij}^{+}\Delta lnGE_{i,t-j}^{\;\;\;\;\;\;\;\;\;+}+\phi_{ij}^{-}\Delta lnGE_{i,t-j}^{\;\;\;\;\;\;\;\;\;-})\;+\sum_{k=0}^{u-1}(\varphi_{ik}^{+}\Delta lnGDP_{i,t-k}^{\;\;\;\;\;\;\;\;\;+}\\ & +\varphi_{ik}^{-}\Delta lnGDP_{i,t-k}^{\;\;\;\;\;\;\;\;\;-})+\sum_{l=0}^{v-1}(\psi_{il}^{+}\Delta lnURB_{i,t-l}^{\;\;\;\;\;\;\;\;\;+}+\psi_{il}^{-}\Delta lnURB_{i,t-l}^{\;\;\;\;\;\;\;\;\;-})\\ & +\sum_{m=0}^{w-1}(\lambda_{im}^{+}\Delta lnHC_{i,t-m}^{\;\;\;\;\;\;\;\;\;+}+\lambda_{im}^{-}\Delta lnHC_{i,t-m}^{\;\;\;\;\;\;\;\;\;-})+\theta_{1}lnEFP_{i,t-1}\\ & +\theta_{2}^{+}\Delta lnREC_{i,t-1}^{+}+\;\theta_{3}^{-}\Delta lnREC_{i,t-1}^{-}+\theta_{4}^{+}\Delta lnNRR_{i,t-1}^{+}+\theta_{5}^{-}\Delta lnNRR_{i,t-1}^{-}\\ & +\;\theta_{6}^{+}\Delta lnGE_{i,t-1}^{+}+\theta_{7}^{-}\Delta lnGE_{i,t-1}^{-}+\;\theta_{8}^{+}\Delta lnGDP_{i,t-1}^{+}+\theta_{9}^{-}\Delta lnGDP_{i,t-1}^{-}\\ & +\theta_{10}^{+}\Delta lnURB_{i,t-1}^{+}+\theta_{11}^{-}\Delta lnURB_{i,t-1}^{-}+\;\theta_{12}^{+}\Delta lnHC_{i,t-1}^{+}+\theta_{13}^{-}\Delta lnHC_{i,t-1}^{-}\\ & +\varepsilon_{i,t} \end{array}$$

The error correction model for Equation (13) is as follows:

(14) $$\begin{array}{cc} \Delta lnEFP_{it}=\alpha_{0} & +\sum_{d=1}^{p-1}\beta_{id}\Delta lnEFP_{i,t-d}+\sum_{f=0}^{r-1}(\gamma_{if}^{+}\Delta lnREC_{i,t-f}^{\;\;\;\;\;\;\;\;\;+}+\gamma_{if}^{-}\Delta lnREC_{i,t-f}^{\;\;\;\;\;\;\;\;\;\;\;-})\\ & +\sum_{q=0}^{s-1}(\delta_{iq}^{+}\Delta lnNRR_{i,t-q}^{\;\;\;\;\;\;\;\;\;\;\;+}+\delta_{i}^{-}\Delta lnNRR_{i,t-q}^{\;\;\;\;\;\;\;\;\;\;-})\\ & +\sum_{j=0}^{t-1}(\phi_{ij}^{+}\Delta lnGE_{i,t-j}^{\;\;\;\;\;\;\;\;\;+}+\phi_{ij}^{-}\Delta lnGE_{i,t-j}^{\;\;\;\;\;\;\;\;\;-})\;+\sum_{k=0}^{u-1}(\varphi_{ik}^{+}\Delta lnGDP_{i,t-k}^{\;\;\;\;\;\;\;\;\;+}\\ & +\varphi_{ik}^{-}\Delta lnGDP_{i,t-k}^{\;\;\;\;\;\;\;\;\;-})+\sum_{l=0}^{v-1}(\psi_{il}^{+}\Delta lnURB_{i,t-l}^{\;\;\;\;\;\;\;\;\;+}+\psi_{il}^{-}\Delta lnURB_{i,t-l}^{\;\;\;\;\;\;\;\;\;-})\\ & +\sum_{m=0}^{w-1}(\lambda_{im}^{+}\Delta lnHC_{i,t-m}^{\;\;\;\;\;\;\;\;\;+}+\lambda_{im}^{-}\Delta lnHC_{i,t-m}^{\;\;\;\;\;\;\;\;\;-})+\eta ECT_{i,t-1}+\varepsilon_{i,t} \end{array}$$

$\gamma_{if}^{+}$, $\gamma_{if}^{-}$, $\delta_{iq}^{+}$, $\delta_{iq}^{-}$, $\phi_{ij}^{+}$, $\phi_{ij}^{-}$, $\varphi_{ik}^{+}$, $\varphi_{ik}^{-}$, $\psi_{il}^{+}$, $\psi_{il}^{-}$, $\lambda_{im}^{+}$, and $\lambda_{im}^{-}$ are short-run asymmetry dynamics, and *η* is the coefficient of the error correction term.

We will estimate the NARDL model with the pooled mean group (PMG) method. Hausman test will also be applied to ascertain the validity of the PMG estimator. The Wald test will be used to determine the existence of an asymmetric relationship between the variables.

## 4. Empirical Results and Discussion

### 4.1. Descriptive Statistics

A summary of the descriptive statistics of the variables is displayed in Table 2. At a glance, we can tell that none of the variables in the groups is normally distributed. The skewness values have to be 0 for normal distribution and kurtosis 3. Natural resource rents are negatively skewed in all the groups, while government effectiveness is negatively skewed in two groups (the whole sample and countries with ecological deficits) but positively skewed in the group of countries with ecological reserves. Mean statistics show an average GDP of 23.505 for the whole sample. Countries with ecological deficits have a higher average GDP (23.672) than countries with ecological reserves (23.222). We also observed that average renewable energy consumption (4.211), urbanization (3.771), and natural resource rents (2.014) are higher in countries with ecological reserves than in countries with ecological deficits.

From the analysis of the descriptive statistics above, we can infer that those countries with ecological reserves have a higher economic growth (GDP) and renewable energy consumption than countries with ecological deficits. The group of countries with ecological deficits, on the other hand, has a higher rate of government effectiveness index and natural resource rents.

### 4.2. Correlation and Variance Inflation Factor

The purpose of the correlation and variance inflation factor (VIF) analysis is to check for multicollinearity among the variables, which might lead to spurious regression and incorrect estimates. The results for correlations and VIF are presented in Table 3. Correlation results show that REC is negatively correlated with EFP in the whole sample and both subgroups. This shows REC has a negative relationship with EFP. URB and HC are negatively correlated with EFP in the whole sample and ecological deficit countries but positively correlated with EFP in ecological reserve countries. NRR has a positive relationship with EFP in the whole sample but is negative in the two subsamples. GE is positively correlated with EFP in all groups. The results of VIF are all below ten, which indicated that there is no problem of multicollinearity among the variables.

### 4.3. Cross-Sectional Dependence Test

Ubiquitous cross-sectional dependency can occur in panel data, where all variables in one cross-section are associated. This is generally related to the effects of some unobserved explanatory variables that are identical to all groups and influence them in distinct ways. Most panel data models are estimated under the assumption that observations made by different participants are unrelated [74]. Theoretically, economic variables are often expected to engage in behavior that creates dependency on one another. The presumption of cross-sectional independence may lead to inconsistent estimations if the variables are interdependent. It is important not to ignore or take for granted the fact that the countries studied have many common characteristics and homogeneities [75]. For this reason, it is necessary to perform a preliminary test for cross-sectional dependence before estimating the model. The results of a cross-sectional dependence test will also help us decide whether to use first-generation unit root tests or second-generation unit root tests. We performed the Breusch–Pagan Lagrange multiplier (LM), Pesaran scaled Lagrange multiplier (LM), bias-correlation scaled Lagrange multiplier (LM), and the Pesaran cross-sectional dependence (CD) tests to check for cross-sectional dependence in our data. Cross-sectional dependence is tested under the null hypothesis of no cross-sectional dependence (H0 = no cross-sectional dependence). Results in Table 4 show that the null hypothesis of no cross-sectional dependence is rejected in all the panels (whole sample, countries with ecological deficits, and countries with ecological reserves). We therefore conclude that there is cross-sectional dependence in our data. This suggests the presence of considerable interconnectedness and dependencies between the African economies. In the presence of cross-sectional dependence, it is appropriate to conduct second-generation panel unit root tests.

### 4.4. Unit Root Test

Unit root tests are necessary to determine the order of integration of the variables and avoid the likelihood of obtaining spurious estimates. We conducted unit root tests using Pesaran’s [76] cross-sectionally augmented IPS (CIPS). Pesaran’s CIPS test is robust in the presence of cross-sectional dependence and heterogeneity. We tested the variables for unit root with constant and constant and trend in our estimation. The results of CIPS unit root tests in Table 5 reveal that for the whole sample, all the variables except for lnNRR are non-stationary at the level with constant, but with constant and lnEFP, lnNRR, and lnGE are stationary at the level. After taking the first difference, all variables in the whole sample became stationary. Therefore, the whole sample includes variables of both I (0) and I (1) orders of integration. In the group of countries with ecological deficits, all variables are integrated of I (1) with a high statistical significance of 1% after first differencing. Similar to the whole sample, the panel of countries with ecological reserves showed that variables are integrated of both I (0) and I (1). In both the whole sample and the panel of countries with ecological reserves, lnNRR is the only variable that was stationary at the level with constant and trend.

Second-generation CIPS unit root tests showed that the variables used in our study are a mixture of I (0) and I (1) variables. This meets the requirements for the estimation of ARDL and NARDL models. Before estimating the model, we test for cointegration among the variables in all the panels.

### 4.5. Westerlund Cointegration Test

Cointegration is pivotal in analyzing the long-run associations in times series data. In this study, we conduct a cointegration test to determine whether there is a long-run relationship among our variables. There are many approaches to checking for cointegration; each has its advantages and could provide distinct outcomes. We employed the Westerlund–Edgerton [77] bootstrap test of cointegration in this study because it accounts for cross-sectional dependence. Empirical evidence based on the results in Section 4.2 showed there is cross-sectional dependence in our data, giving us enough justification to use the Westerlund–Edgerton cointegration test for the null hypothesis of no cointegration. Based on the output in Table 6, Ga and Pa statistics do not reject the null hypothesis of no cointegration, but Gt and Gt statistics strongly reject the null hypothesis. We therefore reject the null hypothesis of no cointegration for all the panels in our study. All variables in each panel are cointegrated, and a long-run relationship exists. We can therefore proceed to run our ARDL and NARDL models.

### 4.6. The Symmetric ARDL Results

Having confirmed that all the variables are cointegrated in all the groups under study, we proceed to estimate the PMG ARDL model. The optimum number of lags was determined using the Akaike information criteria (AIC). The optimum lags for the ARDL model are (1, 1, 1, 1, 1, 1, 1). Short-run and long-run coefficients of the variables are presented in Table 7, and interpretations are given below:

- Whole sample

The findings show a negative relationship between lnREC in short and long runs for the whole sample. The long-run coefficient of lnREC (−0.120) is significant at 1%, while the short-run coefficient (−0.433) is significant at 10%. This implies that a 1% increase in renewable energy consumption leads to a 0.12% decrease in ecological footprint in the long term and a 0.43% decrease in the short term. In line with our findings, authors of Refs [2,10,11,78] also found a negative relationship between renewable energy consumption and ecological footprint. This proves that increasing renewable energy consumption will improve the quality of the environment [8,26,36] and reduce the ecological footprint of African countries. Long-run estimates of lnNRR and lnGDP are positive in the long run and short run. We deduce that 1% in natural resource rents will increase ecological footprint by 0.04% in the long run, with a statistical significance of 1%. In the short run, an increase in natural resource rents increases ecological footprint by 0.02%, with a 5% significance. An increase in natural resource rents trickles down to the rise in natural resource extraction, which will cause harm to the environment [2,60,63,79]. Similarly, in the long run, a one percent increase in the gross domestic product will cause an increase in the ecological footprint of African countries by 0.11%. An increase in economic growth is detrimental to the environment [8,10,80]. lnGE (0.036) has a significantly positive effect on lnEFP in the long run. Government effectiveness is expected to increase ecological footprints; this clearly shows the need for African governments to take massive measures to ensure sound and sustainable implementation of policies. The positive association between economic growth and ecological footprint is in line with the findings of Refs [11,81,82,83,84]. Economic growth degrades the environment in the long run [85]. This proves that Africa is pursuing economic growth to the detriment of the environment [15].

lnURB (−0.686) and lnHC (−1.957) have had a positive impact on lnEFP in the long run, at a high statistical significance of 1%. Similarly, a 1% increase in urbanization and human capital will decrease ecological footprint by 0.69% and 1.96%, respectively. According to Danish et al. [10], Charfeddine and Mrabet [31], and Nathaniel et al. [85] urbanization promotes the quality of the environment in the long run. In contrast, Clancy [9], Wang and Dong [15], and Al-Mulali et al. [58] found that urbanization worsens the quality of the environment. Human capital has the biggest negative impact (−1.96) on the ecological footprint in the long run. Zafar et al. [66] and Mensah et al. [12] also found a negative effect of human capital on the environment, while Danish et al. [19] found no relationship between human capital and ecological footprint. The coefficient of the error correction term for the whole sample shows a 59% speed of adjustment to long-run equilibrium yearly. Hausman’s test results back the use of the PMG estimator over the others;

- Countries with Ecological Deficits

In the group of countries with ecological deficits, long-run and short-run estimates of lnREC are similar to the results of the whole sample. lnREC has a negative impact of 0.17% on lnEFP in the long run and −0.87% in the short run. lnNRR exhibits a positive relationship with lnEFP, at a significance of 5% in the long run. A percentage increase in natural resource rents is expected to increase ecological footprint by 0.035 in countries with ecological deficits. An increase in natural resource rents is unfavorable for the environment [12]. lnGDP displays a positive relation with lnEFP in both the short and long run. Coefficient estimates show that a 1% percent increase in GDP will cause a 0.21% increase in EFP in the long run and a 0.61% increase in the short run in countries with ecological deficits. Government effectiveness has no impact on the ecological footprint in this group; both long-run and short-run coefficients of lnGE are statistically insignificant. Urbanization significantly reduces ecological footprint by 0.42% in the long run but has no impact in the short run. Human capital in countries with ecological deficits has adverse effects on the ecological footprint in the short and long run. However, long-run estimates are statistically insignificant. In essence, a 1% increase in human capital will cause a decrease of 8.5% in ecological footprint. The error correction term reports an 87% speed of adjustment to long-run equilibrium yearly. Hausman test results support the use of the PMG ARDL model;

- Countries with Ecological Reserves

Similar to the whole sample and the group of countries with ecological deficits, renewable energy consumption reduces the ecological footprint of countries with ecological reserves in the long run and the short run. There is no statistical evidence to back the impact of lnNRR on lnEFP in this group. Results in Table 7 demonstrate that lnNRR has a positive relationship with lnEFP in the long run and a negative association in the short run, but estimates are statistically insignificant. This means the natural resource rents do not influence the quality of the environment in countries with ecological reserves. Findings on the impact of lnGDP show that, in contrast to the first two groups, GDP has a 0.24% negative effect on lnEFP in the long run. This implies that economic growth does not harm the quality of the environment in countries with ecological reserves. In the short run, results are similar to the other two groups, where GDP positively impacts lnEFP. The long coefficients of lnURB (−1.299) and lnHC (−3.541) show that a percentage increase in urbanization and human capital has a 1.3% and 3.5% effect on ecological footprint reduction in countries with ecological reserves, a 1% level of significance. However, lnURB (15.715) and lnHC (13.673) are positive but insignificant in the short run. On the other hand, government effectiveness significantly increases ecological footprint by 0.27% in the long run but has an insignificant decreasing effect in the short run. The coefficient is negative and significant, showing a 52% adjustment to long-run equilibrium. Hausman test results agree with the use of the PMG ARDL model.

Comparatively, the results in Table 7 show that renewable energy consumption has a negative impact on the ecological footprint in both the long run and the short run in both the whole sample and the subgroups. Natural resource rents are also seen to have positive and significant relation with lnEFP in the long run in all the groups. A resource-rich continent like Africa should benefit from the positive impacts of natural resource abundance. However, the strain placed on natural resources to meet rising energy and growth demands has put much pressure on the environment. Ideally, Africa should use its abundant renewable energy resources to cut down on fossil energy use and imports. The findings of this study suggest unsustainable resources are a leading factor in environmental degradation in Africa. We find that GDP has positive impacts on all groups. According to the findings, African economies are growing while having insufficient environmental protection laws, diminishing the link between economic development and environmental degradation. Furthermore, Africa’s fundamental production methods are more generally nonrenewable and energy intensive, resulting in increased economic expansion without regard for environmental quality. Results further highlight the importance of urbanization and human capital in achieving environmental sustainability.

### 4.7. The Asymmetric (Nonlinear) ARDL Results

We proceed to estimate the NARDL model using the partial sum decomposition of each dependent variable to test for the impacts of each variable’s negative and positive shocks on ecological footprint. Using the Akaike information criteria (AIC), the optimum lags for the NARDL model are (1, 1, 1, 1, 1, 1, 1). Table 8 presents the asymmetric long-run and short-run estimates of the whole sample and the subsamples (countries with ecological deficits and countries with ecological reserves).

- Whole sample

In the total sample, the decomposed values of lnREC (lnREC+ and lnREC−) show that in the long and short run, lnREC+ has a significantly negative effect of 0.1% and 0.2% on lnEFP. lnREC−, on the other hand, only has a significantly positive impact of 0.11% on lnEFP in the long run. Renewable energy consumption has a good effect on the environment, as it reduces ecological footprints presently and in the future [11]. Both positive and negative shocks of natural resource rents have insignificant impacts on the ecological footprint in the long and short run. Positive and negative shocks of GDP significantly increase ecological footprints in the short and long run. Estimates show that a 1% increase in lnGDP+ (lnGDP−) will lead to a 0.2% (0.124) increase in EFP in the long run. Mujtaba et al. [11] also found a positive relationship between the positive shocks of economic growth and ecological footprint. Positive shocks of urbanization significantly reduce ecological footprints by 0.2% in the long run and 0.8% in the short run. Positive (0.39) and negative shocks (0.39) of government effectiveness positively affect the ecological footprint in the long run, and estimates are statistically significant. Only the positive shock of human capital is positive and significant in the short run at a significance level of 10%;

- Countries with Ecological Deficits

In countries with ecological deficits, positive shocks of renewable energy consumption have insignificantly negative impacts on the ecological footprint in the long and short run. Negative shocks of renewable energy, on the other hand, have a 0.04% negative impact on ecological footprint at a significance level of 5% in the long run and no significant effect in the short run. lnNRR+ and lnNRR− have a negative influence of 0.2% and 0.03% on ecological footprint in the short run and a positive impact of 0.1% and 0.11% in the long run. Positive and negative shocks of GDP have a positive and significant association with lnEFP in both the long and short run. Positive shocks of GDP increase EFP by 0.12% in the long run and 0.09% in the short run. At the same time, the negative shocks of GDP increase EFP by 0.15% in the long run and 0.48% in the short run. Coefficient estimates of lnGE+ and lnGE− are insignificant in the short and long run. This implies that government effectiveness does not affect the ecological footprint of countries with ecological deficits. Positive shocks of urbanization reduce ecological footprint by 0.1% in the long run and 5.8% in the short run. The negative shocks of urbanization significantly decrease ecological footprint by 0.34% in the short run. We also find that positive shocks of human capital reduce the ecological footprint by 0.67% in the long and 5% in the short run. Negative shocks of human capital decrease EFP significantly by 2.9% in the long run;

- Countries with Ecological Reserves

In countries with ecological reserves, lnREC+ and lnREC− reduce ecological footprint in the long run by 0.2% and 0.1%, respectively, at a statistical significance of 1%. In the short run, lnREC+ (0.029) and lnREC− (0.032) have positive but insignificant effects on ecological footprint. lnNRR+ and lnNRR− have a significantly negative relation with ecological footprint in the long but a positive nonsignificant relation in the short run. Both lnNRR+ and lnNRR− have a 0.18 % negative impact on EFP in the long run. lnGDP+ (0.08) and lnGDP− (0.09) have a negative effect on the ecological footprint in the long run and a positive insignificant effect in the short run. lnGE+ and lnGE− have significantly positive effects in both the long and short run. lnGE+ increased EFP by 0.41% and 0.56% in the long and short run, respectively. lnGE is estimated to cause a 0.33% and a 0.55% rise in EFP in the long and short run. lnURB+ has a 0.4% negative and significant impact on EFP in the long run but has an insignificant influence in the short run. lnURB-, on the other hand, is insignificant in both the long and short run. lnHC+ (−0.44) and lnHC− (−2.5) are negative and significant in the long run but insignificant in the short run.

The error correction terms of all three groups are negatively significant and less than one, indicating a 50%, 40%, and 41% yearly speed of adjustment to equilibrium in the whole sample (countries with ecological deficits and countries with ecological reserves, respectively). All Hausman test results support the use of the PMG estimator.

### 4.8. Wald Tests

Wald test results in Table 9 prove the existence of long-run asymmetry in almost all the variables in all three groups. All variables except GDP show statistical evidence of an asymmetric relationship in the whole sample group. We can infer that renewable energy consumption, natural resource rents, government effectiveness, urbanization, and human capital have a significant nonlinear relationship with environmental sustainability in African countries in the long run. Results also show that in countries with ecological deficits, there is no evidence of a nonlinear relationship between renewable energy consumption and ecological footprint. However, lnNRR, lnGDP, lnGE, and lnURB show the existence of asymmetry in the long and short run in countries with ecological deficits. Countries with ecological reserves display statistically significant evidence of a nonlinear relationship between ecological footprint and all the independent variables in the long run.

## 5. Conclusions

Environmental degradation has grave consequences for living beings, as biodiversity declines. Increasing environmental sustainability evaluation is one strategy to assist African countries in achieving sustainable development goals. The paper concentrates on Africa because of the diversity of its countries, some of which have ecological deficits and others, which have surpluses. This enables a comparison of the two groups’ ecological sustainability. This research examines and expands the literature on environmental sustainability and ecological footprint and the importance of government effectiveness in African countries, which has received little attention. The present study varies from earlier research; it explores both symmetric and asymmetric influences of variables on the ecological footprint in two subgroups (countries with ecological deficits and countries with ecological reserves). It provides more detailed evidence of the link between natural resources, green energy consumption, urbanization, human capital, government effectiveness, and ecological sustainability in African countries from 1990 to 2018. The study examines cross-sectional dependencies before using a second-generation panel unit root test that accounts for cross-sectional dependence. Cointegration is also confirmed with the Westerlund bootstrap cointegration, which accounts for cross-sectional dependence. After confirming the existence of a cointegration relationship, the study performs the symmetric and asymmetric panel ARDL model to test for long-run coefficient estimates. Furthermore, the Wald test confirms the existence of long-run asymmetry in the nonlinear panel ARDL estimations.

The study’s main findings show that renewable energy consumption significantly reduces the ecological footprint by 0.12% in African countries in the long run. Asymmetric estimates show that positive shocks (−0.17) and negative shocks (−0.13) of renewable energy consumption significantly reduce ecological footprints in countries with ecological reserves in the long run. In countries with ecological deficits, negative shocks of renewable energy and consumption have a negative effect of 0.04% on the ecological footprint. Natural resources are partly to blame for rising ecological footprints in African countries. However, while natural resources rent reduces ecological footprints in countries with ecological deficits, it has no impact on the environment of countries with ecological reserves. The partial sum decomposition of variables shows that positive shocks in natural resource rents increase ecological footprint by 0.1% in countries with ecological deficits and reduce EFP by 0.18% in countries with ecological reserves in the long run. Economic growth proved to be a driver of environmental degradation in Africa and the subgroups. Both positive and negative shocks of GDP raise ecological footprints in the long run. In general, urbanization and human capital contribute significantly to environmental sustainability. Government effectiveness diminishes environmental sustainability in Africa as a whole and countries with ecological reserves over time. However, government effectiveness has no impact on the environment in countries with ecological deficits.

Our findings further reveal that renewable energy consumption, natural resource rents, government effectiveness, urbanization, and human capital have a significant nonlinear relationship with environmental sustainability in African countries in the long run. Results show that in countries with ecological deficits, there is no evidence of a nonlinear relationship between renewable energy consumption and ecological footprint. However, lnNRR, lnGDP, lnGE, and lnURB show the existence of asymmetry in the long and short run in countries with ecological deficits. Countries with ecological reserves display statistically significant evidence of a nonlinear relationship between ecological footprint and all the independent variables in the long run.

### 5.1. Policy Recommendations

The study’s findings suggest several policy implications that might assist governments and policymakers in the African nations studied in reducing the negative consequences of environmental problems. The long-run relationship between the variables studied indicates the particular relevance of renewable energy consumption, urbanization, human capital, economic growth, and ecological footprint to the environmental sustainability of African countries. Per our analysis, it is advised that African governments should encourage investment in renewable energy technologies that can help with climatic change mitigation strategies to meet sustainable development goals. Including renewable energy in the energy mix may reduce the continent’s ecological footprint. The government must take initiatives to make transportation more energy efficient and environmentally friendly. Furthermore, in Africa, where natural resources abound, biofuel and solar energy might be a significant step toward reducing reliance on nonrenewable energy. Based on the evidence that renewable energy enhances environmental quality, authorities should prioritize producing and using renewable energy sources. Businesses and industries that create and use renewable energy should be given special incentives and rewards. Renewable energy generation might be achieved by increasing investments in research and development investment on the continent. The government should encourage public–private partnerships to raise environmental awareness and the acceptability of alternative energy technologies in society. The present study calls for developing more contemporary, effective, and environmentally friendly renewable energy sources to maintain environmental sustainability. Adopting sustainable practices in the natural resource industry by increasing low-polluting energy sources is one potential method to achieve ecological sustainability and reduce ecological footprints. The increased demand for natural resources and their availability is a major concern for both ecological deficit and ecological reserve countries. A shift in mindset is necessary, with a better understanding of the demand for sustainable development in all countries; otherwise, environmental deterioration would impede the advancements that the economy requires. Excellent governance and strategic planning should regulate the unsustainable use of natural resources. Because institutional responsibility is so important, a country’s central entities should promote accountability and openness for sustainable natural resource use. Furthermore, environmentally friendly technology in resource extraction should be promoted. In this regard, the study advises that policymakers encourage the efficient and sustainable use of natural resources and an increase in the use of clean and sustainable energy to achieve higher environmental quality and long-term sustainable growth. Human capital development is also required for the protection of the environment. Governments of African countries should increase expenditure on health and education. It is widely known that investing in education and healthcare is a fundamental way for a country to improve its human capital stock; consequently, increasing public investments in human capacity development is critical for raising awareness about preserving environmental health. This will create an avenue for individuals and businesses to learn about recycling, conservation, cleaner production, and responsible citizenship. Countries with ecological deficits, such as Mauritius, Kenya, South Africa, Egypt, Algeria, Tunisia, and Morocco, have misused their ecological resources in the quest for high economic growth that would allow them to advance and create better prospects in their societies. They must all introduce technological advancements that would enable them to thrive while keeping a healthy relationship with the natural resources they exploit. To put it another way, countries with ecological deficits must adopt sustainable practices and energy-saving measures. Gabon stands out among the countries with ecological reserves because of its unique behavior patterns. Gabon is one of the African countries that has experienced rapid economic expansion while making good use of its forests. Gabon has reduced forest-related emissions while preserving its forests and staying within its environmental limits. We attribute this to the intellectual maturity among its citizens and government effectiveness, which has enabled the country to ensure that the appropriate use of natural resources accompanies its progress. The instance of Gabon should serve as a model for other African countries seeking environmental sustainability. To summarize, Africa faces two challenges: first, developing policies and programs to mitigate the impact of increasing crisis and price of natural capital on the welfare of its people; and second, working with the rest of the globe to slow and ultimately restore overall ecological balance. Authorities need to accomplish rapid economic expansion, plan urbanization, and support the renewable energy sector, while also considering environmental health. To achieve a truly sustainable economic development, all member nations must reinforce country-specific and regional environmental accords, such as the Paris Agreement, the Kyoto Protocol, and the Sustainable Development Goals (SDGs) of the United Nations.

### 5.2. Limitations

The conclusions reached are not without limits due to the methods used. A change in the sample analyzed and the addition of variables could influence the outcomes. The lack of a large dataset restricted the study, and some other determinants of environmental sustainability, such as foreign direct investment, financial development, trade openness, globalization, and technology, were not considered. The results are based on Africa; a comparison of African and Asian countries, on the other hand, would bring a new dimension to the research. Future research should look into the environmental Kuznets curve and the pollution halo–heaven hypotheses concerning the necessity to transition to renewable energy sources. These theories would support the need for a shift to a more sustainable approach.

## Figures and Tables

**Table 1:** Description of variables.

Variable	Description	Unit of Measurement	Source
EFP	Ecological Footprint	Global hectares per capita	Global Footprint Network (2022) https://data.footprintnetwork.org/#/ (accessed on 23 March 2022)
REC	Renewable Energy Consumption	Renewable Energy Consumption (% total final energy consumption)	World Development Indicators (WDI, 2022) https://databank.worldbank.org/source/world-development-indicators (accessed on 23 March 2022)
NRR	Natural Resource Rents	Natural Resource rents (% of GDP)	World Development Indicators (WDI, 2022) https://databank.worldbank.org/source/world-development-indicators (accessed on 23 March 2022)
GDP	Gross Domestic Product	Constant 2015 USD	World Development Indicators (WDI, 2022) https://databank.worldbank.org/source/world-development-indicators (accessed on 23 March 2022)
GE	Government Effectiveness	A measure of the quality of governance with values ranging from −2.5 (weak) to 2.5 (strong)	Worldwide Governance Indicators (WGI, 2022) https://databank.worldbank.org/source/worldwide-governance-indicators (accessed on 23 March 2022)
URB	Urbanization	Urban Population (% of the total population)	World Development Indicators (WDI, 2022) https://databank.worldbank.org/source/world-development-indicators (accessed on 23 March 2022)
HC	Human Capital	Human Capital Index (based on years of schooling and returns to education)	Penn World Table 10.0 https://www.rug.nl/ggdc/productivity/pwt/ (accessed on 12 April 2022)

**Table 2:** Descriptive statistics of variables.

	Mean	Std. Dev.	Minimum	Maximum	Skewness	Kurtosis
Whole Sample						
lnEFP	0.328	0.415	−0.446	1.513	0.564	2.737
lnREC	3.742	1.238	−2.830	4.588	−2.706	11.335
lnNRR	1.715	1.612	−6.750	4.023	−2.554	11.485
lnGDP	23.505	1.486	20.689	26.922	0.349	2.328
lnGE	1.053	0.243	0	1.522	−0.592	3.610
lnURB	3.547	0.550	1.689	4.493	−0.888	3.347
lnHC	0.541	0.239	0.029	1.069	0.012	2.208
Countries with Ecological Deficits						
lnEFP	0.329	0.414	0.446	1.513	0.599	2.955
lnREC	3.466	1.46	−2.830	4.573	−2.065	7.386
lnNRR	1.540	1.823	−6.750	3.701	−2.580	9.904
lnGDP	23.672	1.693	20.689	26.921	0.196	1.819
lnGE	1.094	0.238	0	1.522	−1.094	5.291
lnURB	3.415	0.612	1.689	4.285	0.600	2.377
lnHC	0.560	0.234	0.029	1.049	−0.277	2.301
Countries with Ecological Reserves						
lnEFP	0.327	0.416	−0.410	1.311	0.504	2.372
lnREC	4.211	0.373	3.308	4.588	−1.248	3.125
lnNRR	2.014	1.110	−0.717	4.023	−0.353	2.598
lnGDP	23.222	0.982	21.052	25.479	−0.137	2.948
lnGE	0.983	0.236	0.494	1.447	0.161	2.360
lnURB	3.771	0.317	3.149	4.493	0.367	2.667
lnHC	0.510	0.244	0.097	1.069	0.476	2.391

**Table 3:** Correlation and VIF results.

	lnEFP	lnREC	lnNRR	lnGDP	lnGE	lnURB	lnHC	VIF
Whole Sample								
lnEFP	1.0000							
lnREC	−0.5291	1.0000						1.52
lnNRR	0.4595	0.1603	1.0000					1.64
lnGDP	0.2844	−0.4825	0.0918	1.0000				1.67
lnGE	0.6560	−0.3785	−0.5741	0.2445	1.0000			2.35
lnURB	−0.5744	−0.4440	−0.0811	0.5273	0.3346	1.0000		1.63
lnHC	−0.6705	−0.4121	−0.3312	0.3244	0.6362	0.4561	1.0000	1.93
Countries with Ecological Deficits								
lnEFP	1.0000							
lnREC	−0.5933	1.0000						1.76
lnNRR	−0.4429	0.0786	1.0000					1.61
lnGDP	0.5140	−0.5148	0.0835	1.0000				2.21
lnGE	0.6109	−0.3094	−0.5366	0.3211	1.0000			2.35
lnURB	−0.7227	−0.6424	−0.2120	0.6706	0.5322	1.0000		2.48
lnHC	−0.6512	−0.4293	−0.3278	0.4714	0.6379	0.5359	1.0000	2.03
Countries with Ecological Reserves								
lnEFP	1.0000							
lnREC	−0.6910	1.0000						2.13
lnNRR	−0.5691	0.6517	1.0000					2.18
lnGDP	−0.3695	−0.0185	0.2943	1.0000				1.44
lnGE	0.7703	−0.7856	−0.6973	−0.0640	1.0000			2.14
lnURB	0.3489	−0.2296	0.2626	0.3564	0.2187	1.0000		2.57
lnHC	0.7116	−0.5629	−0.3397	−0.0810	0.6164	0.6133	1.0000	2.46

**Table 4:** Cross-sectional dependence test results.

	Breusch–Pagan LM		Pesaran Scaled LM		Bias-Correlation Scaled LM		Pesaran CD	
	Statistic	Prob.	Statistic	Prob.	Statistic	Prob.	Statistic	Prob.
Whole Sample								
lnEFP	3229.072 *	0.000	108.625 *	0.000	108.144 *	0.000	2.631 *	0.009
lnREC	3427.181 *	0.000	116.103 *	0.000	115.621 *	0.000	40.727 *	0.000
lnNRR	1580.444 *	0.000	46.402 *	0.000	45.920 *	0.000	15.711 *	0.000
lnGDP	8503.098 *	0.000	307.681 *	0.000	307.199 *	0.000	91.274 *	0.000
lnGE	1308.296 *	0.000	36.131 *	0.000	35.420 *	0.000	−0.357	0.721
lnURB	9044.790 *	0.000	328.126 *	0.000	327.644 *	0.000	73.760 *	0.000
lnHC	8852.574 *	0.000	320.871 *	0.000	320.389 *	0.000	89.733 *	0.000
Countries with Ecological Deficits								
lnEFP	1165.240 *	0.000	62.407 *	0.000	62.130 *	0.000	3.520 *	0.000
lnREC	1621.255 *	0.000	90.057 *	0.000	89.753 *	0.000	33.562 *	0.000
lnNRR	612.120 *	0.000	28.869 *	0.000	28.565 *	0.000	89.753 *	0.000
lnGDP	3572.471 *	0.000	208.367 *	0.000	208.063 *	0.000	59.569 *	0.000
lnGE	543.058 *	0.000	24.681 *	0.000	24.234 *	0.000	−1.859 ***	0.064
lnURB	3346.659 *	0.000	194.659 *	0.000	194.354 *	0.000	37.294 *	0.000
lnHC	3335.980 *	0.000	194.227 *	0.000	193.724 *	0.000	53.309 *	0.000
Countries with Ecological Reserves								
lnEFP	414.850 *	0.000	38.981 *	0.000	38.803 *	0.000	−2.295 **	0.027
lnREC	324.498 *	0.000	29.462 *	0.000	29.283 *	0.000	7.021 *	0.000
lnNRR	200.510 *	0.000	16.392 *	0.000	16.214 *	0.000	8.014 *	0.000
lnGDP	925.850 *	0.000	92.849 *	0.000	92.671 *	0.000	29.714 *	0.000
lnGE	122.340 *	0.000	8.152 *	0.000	7.889 *	0.000	1.800 ***	0.071
lnURB	1252.409 *	0.000	127.272 *	0.000	127.093 *	0.000	32.393 *	0.000
lnHC	1186.611 *	0.000	120.336 *	0.000	120.158 *	0.000	34.393 *	0.000

Note *, **, *** represents 1%, 5%, and 10% level of significance, respectively.

**Table 5:** CIPS cross-sectionally dependent unit root test results.

	At Level		First Difference		
	Constant	Constant and Trend	Constant	Constant and Trend	Order of Integration
Whole Sample					
lnEFP	−2.065	−2.917 *	−3.861 *	−4.005 *	I (1)
lnURB	−1.049	−1.939	−2.209 **	−3.455 *	I (1)
lnHC	−1.475	−1.564	−3.151 *	−3.731 *	I (1)
lnNRR	−2.344 *	−2.640 *	−4.487 *	−4.456 *	I (0)
lnREC	−1.804	−2.410	−4.582 *	−4.727 *	I (1)
lnGDP	−1.691	−1.328	−3.611 *	−3.953 *	I (1)
lnGE	−1.897	−2.624 *	−4.425 *	−4.369 *	I (1)
Significance: Constant 1% (−2.33), 5% (−2.17), 10% (−2.08) Constant and trend 1% (−2.84), 5% (−2.68), 10% (−2.60)					
Countries with Ecological Deficits					
lnEFP	−2.584 **	−2.362	−5.506 *	−5.555 *	I (1)
lnURB	−1.988	−2.242	−2.198 *	−2.795	I (1)
lnHC	−1.478	−1.313			I (1)
lnNRR	−1.923	−2.691 ***	−4.932 *	−4.890 *	I (1)
lnREC	−1.517	−2.364	−4.831 *	−4.977	I (1)
lnGDP	−1.839	−1.936	−3.519 *	−3.958 *	I (1)
lnGE	−2.073	−2.869 **	−4.513 *	−4.396 *	I (1)
Significance: Constant 1% (−2.43), 5% (−2.22), 10% (−2.13) Constant and trend 1% (−2.94), 5% (−2.75), 10% (−2.65)					
Countries with Ecological Reserves					
lnEFP	−1.933	−2.813 ***	−5.901 *	−5.746 *	I (1)
lnURB	−1.481	−2.634	−2.755 *	−3.545 *	I (1)
lnHC	−1.251	−2.691	−2.847 *	−3.392 *	I (1)
lnNRR	−2.820 *	−3.353 *	−5.577 *	−5.517 *	I (0)
lnREC	−1.862	−2.051	−4.787 *	−4.707 *	I (1)
lnGDP	−1.908	−1.967	−4.022 *	−4.401 *	I (1)
lnGE	−2.499 **	−2.311	−4.923 *	−4.656 *	I (1)
Significance: Constant 1% (−2.58), 5% (−2.33), 10% (−2.21) Constant and trend 1% (−3.11), 5% (−2.86), 10% (−2.73)					

Note *, **, *** represents 1%, 5%, and 10% level of significance, respectively.

**Table 6:** Results of Westerlund cointegration test.

Statistic	Value	Z-Value	*p*-Value	Robust *p*-Value
Whole sample				
Gt	−3.874	7.773	0.000	0.000
Ga	−6.601	4.310	0.933	0.500
Pt	−19.496	7.692	0.000	0.000
Pa	−9.963	0.450	0.326	0.050
Countries with Ecological Deficits				
Gt	−3.816	5.918	0.000	0.000
Ga	−5.758	3.874	0.963	0.872
Pt	−14.839	5.521	0.000	0.000
Pa	−7.597	0.929	0.824	0.313
Countries with Ecological Reserves				
Gt	−4.098	4.817	0.000	0.000
Ga	−5.287	3.716	1.000	1.000
Pt	−13.626	5.664	0.000	0.000
Pa	−9.325	0.755	0.775	0.200

**Table 7:** PMG ARDL model results.

	Whole Sample	Countries with Ecological Deficits	Countries with Ecological Reserves
Long-run coefficients			
lnREC	−0.120 (0.025) *	−0.173 (0.029) *	−0.201 (0.054) *
lnNRR	0.043 (0.006) *	0.018 (0.007) **	0.013 (0.010)
lnGDP	0.106 (0.040) *	0.208 (0.048) *	−0.239 (0.065) *
lnGE	0.036 (0.020) ***	−0.115 (0.066)	0.265 (0.049) *
lnURB	−0.686 (0.106) *	−0.419 (0.174) **	−1.299 (0.237) *
lnHC	−1.957 (0.129) *	−0.125 (0.106)	−3.541 (0.811) *
Short-run coefficients			
ECT (−1)	−0.593 (0.093) *	−0.866 (0.096) *	−0.523 (0.137) *
∆lnREC	−0.433 (0.245) ***	−0.267 (0.304)	−0.304 (0.210) ***
∆lnNRR	0.024 (0.011) **	−0.002 (0.006)	−0.011 (0.013)
∆lnGDP	0.656 (0.239) ***	0.606 (0.302) **	0.293 (0.157) ***
∆lnGE	−0.143 (0.133)	0.0472 (0.140)	−0.304 (0.263)
∆lnURB	10.789 (18.692)	19.967 (23.028)	15.715 (21.145)
∆lnHC	2.072 (6.224)	−8.590 (5.167) ***	13.673 (11.916)
C	−0.996 (0.491) **	−2.626 (0.563) *	0.925 (0.610) ***
Hausman test	0.59 [0.988]	0.40 [0.998]	3.04 [0.803]

*, **, *** represents 1%, 5%, and 10% level of significance, respectively. () represents the standard error of the estimates. [] represents the probability values of the Hausman test.

**Table 8:** Results of asymmetric ARDL model.

	Whole Sample	Countries with Ecological Deficits	Countries with Ecological Reserves
Long run			
lnREC+	−0.103 (0.017) *	−0.025 (0.018)	−0.165 (0.062) *
lnREC−	0.107 (0.016) *	−0.043 (0.017) **	−0.132 (0.051) *
lnNRR+	0.004 (0.024)	0.103 (0.016) *	−0.179 (0.033) *
lnNRR−	0.019 (0.023)	0.108 (0.015) *	−0.178 (0.038) *
lnGDP+	0.195 (0.079) **	0.123 (0.020) *	−0.086 (0.019) *
lnGDP−	0.124 (0.067) *	0.150 (0.022) *	−0.088 (0.019) *
lnGE+	0.391 (0.044) *	−0.226 (0.186)	0.413 (0.061) *
lnGE−	0.392 (0.045) *	−0.298 (0.187)	0.333 (0.556) *
lnURB+	−0.204 (0.027) ***	−0.101 (0.034) *	−0.404 (0.041) *
lnURB−	−0.498 (0.342)	−1.815 (49.929)	−0.213 (0.021)
lnHC+	0.012 (0.122)	−0.669 (0.037) ***	−0.439 (0.051) *
lnHC−	0.696 (0.635)	−2.916 (0.955) *	−2.502 (0.363) *
Short run			
ECT (−1)	−0.509 (0.063) *	−0.409 (0.079) *	−0.419 (0.078) *
∆lnREC+	−0.015 (0.011) ***	−0.005 (0.221)	0.029 (0.059)
∆lnREC−	−3.013 (0.013)	0.004 (0.019)	0.032 (0.008)
∆lnNRR+	0.012 (0.012)	−0.024 (0.011) **	0.158 (0.027)
∆lnNRR−	0.013 (0.013)	−0.026 (0.012) **	0.032 (0.033)
∆lnGDP+	0.351 (0.158) **	0.089 (0.294) *	0.136 (0.274)
∆lnGDP−	0.344 (0.133) **	0.480 (0.261) ***	0.116 (0.268)
∆lnGE+	−0.144 (0.166)	−0.005 (0.155)	0.563 (0.284) **
∆lnGE−	0.148 (0.144)	0.233 (1.160)	0.555 (0.284) ***
∆lnURB+	−0.822 (5.546) ***	−5.778 (3.551) ***	−10.000 (10.815)
∆lnURB−	0.001 (0.001)	−0.343 (0.252) ***	−0.240 (0.022)
∆lnHC+	0.241 (0.079) ***	−5.009 (2.979) ***	−7.620 (8.964)
∆lnHC−	0.055(0.065)	−2.160 (2.016)	−0.385 (0.038)
C	0.214 (0.010) **	0.209 (0.100) **	0.856 (1.77)
Hausman test	0.55 [0.997]	3.27 [0.514]	0.93 [0.920]

*, **, *** represents 1%, 5%, and 10% level of significance, respectively. () represents the standard error of the estimate. [] represents the probability values of the Hausman test. (+) and (−) represent positive and negative partial sums of variables, respectively.

**Table 9:** Results of Wald tests.

	Whole Sample	Countries with Ecological Deficits	Countries with Ecological Reserves
Long-run asymmetry			
lnREC	7.43 **	2.01	8.03 **
lnNRR	35.04 *	10.64 **	30.37 *
lnGDP	0.34	12.93 *	30.76 *
lnGE	78.01 *	8.35 *	12.07 *
lnURB	43.26 *	8.96 **	9.43 *
lnHC	4.64 ***	9.06 **	39.83 *
Short-run asymmetry			
∆lnREC	0.11	0.05	0.67
∆lnNRR	0.561	3.52 ***	2.44
∆lnGDP	0.32	3.90 **	0.29
∆lnGE	2.76	4.25 **	4.38
∆lnURB	2.75	4.77 (0.92) ***	4.22
∆lnHC	3.95	0.66 (0.416)	0.65

*, **, *** represents 1%, 5%, and 10% level of significance, respectively.

## Data Availability

The data used and their sources are provided in the paper.
